# Perspective: challenges and research opportunities to enhance African Swine Fever control in the Philippines

**DOI:** 10.3389/fvets.2025.1675095

**Published:** 2025-09-30

**Authors:** Chia-Hui Hsu, Rachel A. Schambow, John Humphreys, Maximino Montenegro, Jonathan Arzt, Andres M. Perez

**Affiliations:** ^1^Center for Animal Health and Food Safety, College of Veterinary Medicine, University of Minnesota, Saint Paul, MN, United States; ^2^Agricultural Research Service, National Bio and Agro-Defense Facility, U.S. Department of Agriculture, Manhattan, KS, United States; ^3^Pig Improvement Company (PIC) Philippines, Pasig, Philippines; ^4^Plum Island Animal Disease Center, Agricultural Research Service, U.S. Department of Agriculture, Greenport, NY, United States

**Keywords:** African Swine Fever, research gaps, Philippines, policy, intervention strategies

## Abstract

African Swine Fever (ASF) is recognized as one of the most significant transboundary swine diseases due to its high mortality rate, rapid regional spread, and devastating economic consequences. Since its initial outbreak in July 2019, ASF has afflicted the Philippine swine industry for over 6 years. The implications of the epidemic extend beyond animal health, severely impacting the national pork supply chain and causing environmental concerns. In response, the Philippine government has implemented numerous policies for ASF management and control to support swine industry recovery. However, despite these substantial financial and logistical efforts, ASF remains prevalent, with frequent reemergence in many regions across the country. This perspective paper presents the outcomes of a collaborative workshop with the Philippine College of Swine Practitioners (PCSP), held in Batangas, Philippines, in November 2024. During this initiative, 40 local veterinary experts identified and ranked the most pressing challenges hindering current ASF control. Among the highest-ranked priorities were: (1) the role of intermediaries within the complex swine supply chain, (2) the inconsistent enforcement of guidelines across local government units, and (3) the underreporting of cases due to a lack of relevant local incentives. The resulting prioritizations offer valuable, field-level insights for refining national control strategies and hold significant relevance for neighboring countries facing similar struggles with ASF.

## 1 Introduction

**Figure 1:**
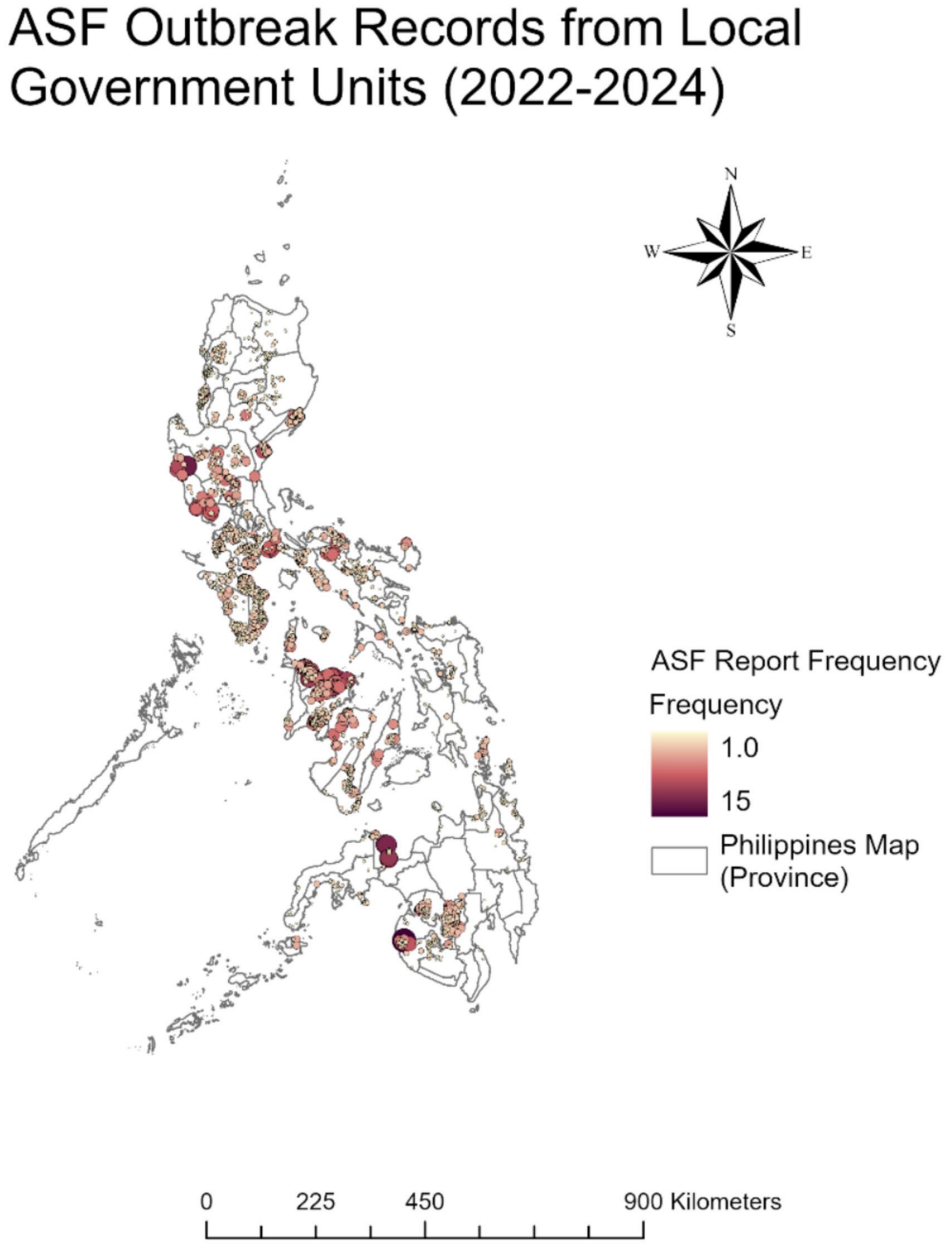
Map showing ASF outbreak report frequency, 2022–2024.

African swine fever (ASF) is a critical concern to the global pig industry and food security due to high disease fatality and associated economic impacts (1). The causal agent, ASF virus (ASFV) is a large DNA virus and sole member of the family *Asfarviridae* (2). In the Philippines, ASF has had considerable economic and social consequences since the first confirmed outbreak in July 2019 (3, 4), with estimated losses reaching around PHP 100 billion (US$1.78 billion) in 2023 (5, 6). Since disease emergence, epidemiological investigations have revealed spatiotemporal clustering of outbreaks, particularly during seasonal peaks from August to October (7), indicating persistent environmental and human-mediated drivers. Despite national efforts, such as routine surveillance updates provided by the Bureau of Animal Industry (BAI) (7), the disease exhibits a recurring pattern (Figure 1), indicating an urgent need for enhanced surveillance, targeted policy interventions, and effective, evidence-based repopulation protocols to support ASF management and pig industry recovery. In response to the widespread impact of ASF, the Philippine government introduced several key programs and policies to control the disease and aid swine population recovery. As summarized by Fernandez-Colorado et al. (8), one of the earliest efforts was Administrative Circular No. 12, Series of 2019, issued by the Philippine Department of Agriculture, which established the National Zoning and Movement Plan to regulate pig movement and safeguard ASF-free zones. This was later followed by the launch of the National ASF Prevention and Control Program (NASFPCP) through Administrative Order No. 07, Series of 2021, led by the Department of Agriculture’s Bureau of Animal Industry, with the objective of combating the spread and impact of ASF and improving local surveillance and farm biosecurity. To complement these efforts, the twin programs Bantay ASF sa Barangay (BABay ASF) for local monitoring and Integrated National Swine Production Initiatives for Recovery and Expansion (INSPIRE) were introduced for repopulation efforts and to help stabilize the hog industry (8–10). Moreover, technical and strategic support has been provided by international partners, including the Food and Agriculture Organization (FAO) through its *Emergency Centre for Transboundary Animal Diseases* in the Asia-Pacific region (11). At the national level, the Philippine Department of Agriculture (DA) has demonstrated its commitment to ASF control by allocating PHP 350 million (US$6.2 million) for vaccine procurement from Vietnam, with the goal of administering 600,000 doses to support and accelerate the national prevention and control efforts in 2024 (12, 13).

Despite these efforts, ASF remains prevalent and continues to re-emerge in many regions across the country. The virus may be transmitted by various mechanisms including direct contact between infected animals, transportation of infected/contaminated pork products, fomites such as equipment, vehicles, and human-mediated activities. This ongoing challenge of ASF in the Philippines raises a critical question: what key obstacles have hindered the effectiveness of ASF eradication and control programs in the Philippines, and what are the opportunities for research activities that might help overcome those challenges?

To address this critical question, we partnered with the Philippine College of Swine Practitioners (PCSP) to design an educational workshop and stakeholder engagement initiative, held in November 2024 in Batangas, Philippines. Through participatory consultations with local stakeholders, government officials at various administrative levels, and field veterinary experts, we examined the current status and challenges of ASF prevention and control, identified key research gaps, and proposed ranking of important issues to enhance future policies and implementation strategies. The insights generated from this initiative are relevant to the Philippine context and hold value for neighboring countries facing similar struggles with ASF in the field.

## 2 Activity development

The Global African Swine Fever Research Alliance (GARA) is an international collaborative network (14), established in 2013, bringing together researchers, institutions, and stakeholders committed to advancing scientific understanding and control strategies for ASF. Through fostering global cooperation and scientific knowledge exchange, GARA is central to coordinating multidisciplinary research efforts and identifying strategic priorities in the fight against ASF. Our methodology was grounded in the gap analysis report published by the GARA in 2022 (15). This document presents a comprehensive synthesis of ASF’s most pressing global knowledge gaps and research priorities, as identified by international expert meetings. The analysis spans multiple domains, including ASF virology, diagnostics, epidemiology, biosecurity practices, vaccine development, and the assessment of control strategies (15).

Building upon this global framework, our work aligns specifically with GARA’s Strategic Goals 1 (identify research opportunities and facilitate collaborations within the Alliance), 2 (conduct strategic and multidisciplinary research to better understand ASF), and 4 (develop novel and improved tools to support the prevention and control of ASF). In pursuit of these goals, we initiated a localized prioritization activity using questionnaire responses from stakeholders in the Philippines.

To localize these efforts, we co-developed a context-specific prioritization activity—revising the global research priority list to reflect the local challenges and practical needs of the Philippine swine industry—in partnership with representatives from the Philippine College of Swine Practitioners (PCSP) and field veterinarians. This prioritization exercise was conducted as part of a capacity-building workshop held in November 2024. We designed and distributed a structured questionnaire using Qualtrics, targeting a total of 40 participants. This group included government officials, field veterinarians, and swine industry experts, all of whom had direct experience with ASF surveillance, diagnostics, outbreak response, or swine production across various regions of the Philippines. The questionnaire was contextually adapted through PCSP expert consultation. This adaptation contributed to the identification and refinement of nine key variables. An open-ended “Other” option was added to capture emerging or context-specific concerns not covered by the predefined categories. The full questionnaire was attached as a Supplementary file. Respondents were asked to rate the importance of each of the nine topics on a Likert scale ranging from 1 (not important at all) to 9 (very important). The responses were analyzed to calculate average importance scores, along with minimum and maximum values and response counts for each variable (see Table 1).

**Table 1:** Ranking results: 9 key variables were developed in collaboration with the PCSP and ranked by 40 local experts during the November 2024 workshop and training program.

Topic	Description	Count	Sum	Mean	Min	Q1	Median	Q3	Max
A	Difficult to get sample from backyard population	40	289	7.23	1	7	7.5	8.5	9
B	Relevant local incentives to report the disease. Underreporting / Policy Non-Compliance	40	320	8	2	7.75	8	9	9
C	Lack of timely information and data sharing between stakeholders.	39	311	7.97	5	7	8	9	9
	Digital epidemiology monitoring and the need to improve traceability.								
D	Inconsistent enforcement of guidelines across LGUs	39	313	8.03	6	7	8	9	9
E	Shifting from Epidemic to an Endemic Mindset	38	270	7.11	3	7	7.5	9	9
F	Urban–Rural Inequality. Limited education and resources for some farmers.	39	273	7	1	6	7	8	9
G	Intermediaries (middleman) is an issue in swine supply chain	39	317	8.13	4	8	9	9	9
H	Inappropriate Slaughtering Practices	36	241	6.69	1	5	7	8	9
I	Vaccine use (will it make ASF control easier or harder?)	36	258	7.17	3	6	8	8	9
J	Others	22	149	6.77	1	6	8.5	9	9

The debriefing workshop and feedback verification constituted the final step of the activity. After the initial data analysis was completed, the results were immediately shared with the participants for validation. During the debriefing session, participants reviewed the statistical findings, provided clarifications, and offered additional insights. This participatory feedback process helped confirm the consensus around key priorities and ensured that the results accurately reflected their field experiences. Ultimately, this debriefing approach strengthened the relevance and credibility of the findings, although in-depth qualitative method or triangulation would be valuable to capture more nuanced perspectives. This participatory approach ensured that the identified priorities reflected ground-level realities. The findings form a foundation for guiding future ASF research, policy development, and intervention strategies in the Philippines.

## 3 Results

As summarized in Table 1, the results reflect expert opinions on the most pressing concerns in ASF management. The issue ranked as most important was the presence of intermediaries (hog traders, also called middlemen or *viajero* in local terms) in the swine supply chain (Mean = 8.13, Median = 9), which was perceived as a significant barrier to effective disease control. The heavy reliance of backyard and small commercial producers on middlemen to sell their pigs, due to their limited direct access to customers, restricts farmers’ bargaining power and access to fair prices. Consequently, they cannot easily bypass these intermediaries to reach consumers or processors directly, impeding effective disease control. This was followed by inconsistent enforcement of guidelines across local government units (LGUs) (Mean = 8.03, Median = 8), where some LGUs implemented quarantine and movement restrictions strictly while others applied them more loosely, leading to gaps in containment. Underreporting of cases due to the absence of relevant local incentives (Mean = 8.00, Median = 8) was also highlighted, as swine farmers often faced financial losses with little or no compensation, discouraging timely reporting. Both of these issues underscore policy and governance limitations. Additionally, a lack of timely data sharing among stakeholders (Mean = 7.97, Median = 8) was identified as a critical challenge, indicating gaps in communication and coordination in veterinary diagnostics and disease surveillance.

During the debriefing workshop discussion, ASF supply chain data and information management emerged as the top priority among the key challenges identified. More specifically, a major concern was the lack of transparency in ASF data collection in the field, particularly regarding the role of middlemen (as highlighted in Table 1, topic G), who are deeply connected in many aspects of the overall swine supply chain. Critical information such as outbreak case reporting at the farm level, slaughter routes, and carcass disposal records was often not clearly documented or readily available. These gaps highlight the urgent need for a centralized and transparent data system to support evidence-based decision-making.

Beyond these immediate issues, the findings also reveal underlying limitations in ASF-related resources. Widespread shortages in both human and financial resources present significant barriers to effective ASF prevention and control. Respondents in the workshop consistently reported a lack of trained personnel (e.g., insufficient lab technicians, diagnostic staff, and field workers), essential logistical support (such as ASF point-of-care testing kits, surveillance tools, and transport), and adequate funding at both national and local administrative levels. These constraints severely hinder rapid ASF outbreak response, sustained long-term surveillance, and the consistent implementation of biosecurity measures. This is especially true for smallholder farms—defined as those with fewer than 20 finishers or 40 piglets—which often lack the resources needed to comply with ASF protocols without external support (16).

Policy implementation and governance issues are closely linked to resource constraints. A key issue identified is the fragmented and inconsistent application of ASF controlling policies across various levels of local government units (LGUs), from regional offices down to the barangay level. While national policies are led by the BAI, their implementation is often delayed or uneven due to the high degree of local autonomy. As several respondents in the workshop noted, “the role of political will varies considerably across administrative regions, leading to disparate local government actions.” This fragmentation is frequently attributed to limitations at the LGU level, including insufficient political commitment, technical expertise, veterinary personnel, and financial capacity to enforce ASF control measures and biosecurity protocols effectively (5, 8). As a result, decision-making often falls to local chief executives, leading to varied local responses that undermine the overall coherence and effectiveness of the national ASF strategy.

The Philippine government is actively promoting agricultural modernization and strengthening ASF control measures. These efforts include nationwide inspections of unlicensed hog farms to ensure compliance with operational and licensing requirements, as well as mentoring programs for backyard farmers (8, 17). However, effective ASF prevention and control often extend beyond government action. Significant challenges within the stakeholder and supply chain networks continue to undermine these efforts. A major obstacle highlighted during the workshop was low stakeholder engagement and continuing sociocultural barriers, which often hinder behavioral change in pig disease control. Similar challenges have been observed in Myanmar, where structural issues impede progress (18). Socioeconomic constraints, such as inadequate indemnification, inconsistent financial support, and limited access to information, further discourage compliance, particularly among resource-limited smallholder or backyard farmers (19). Moreover, farmers, community leaders, and private actors often have limited awareness of ASF’s broader impact (20), contributing to underreporting and poor adherence to control measures (10, 19). To address these challenges, strengthening public-private communication and partnerships will be essential for building trust, aligning stakeholder interests, and enhancing collective action toward effective disease control.

For instance, a particularly pressing LGU issue, which was repeatedly emphasized during the workshop, is the historically low reporting rate of ASF cases, exacerbated by the lack of effective incentives for farmers to report outbreaks. Addressing this challenge requires a multi-faceted approach. First, the reporting system must be clearly defined and standardized to ensure consistent data recording and coordination across all levels of government. Second, indemnification schemes should be sufficiently funded to provide fair compensation for affected farmers, thereby incentivizing timely reporting and collaboration. Third, continuous government mentoring on biosecurity implementation, along with the establishment of stable public-private partnerships, is essential. These elements will help ensure the feasibility of future data collection and maintain stakeholder compliance, ultimately supporting a robust routine surveillance system necessary for effective epidemiological investigations.

## 4 Next, steps in ASF research: key opportunities

In alignment with GARA’s Strategic Goals, this workshop seeks to guide future research direction and inform national policy to enhance the management and control of ASF within the Philippines. The following three priority areas were identified:

Swine supply chain traceability and digital infrastructure: A major gap is the role of middlemen and the lack of clarity around the logistics of the swine supply chain. Establishing clear traceability of swine movement, synchronized documentation across zones, and leveraging existing digital tools is essential to improve transparency and disease control. Currently available digital epidemiology platforms, such as Phil-AHIS—a governmental web-based system that provides statistics, maps, and visualizations of animal disease incident reports (21)—offer valuable opportunities to enhance ASF surveillance and support outbreak response across the country. However, despite the existence of such systems, their utilization remains limited, and their promotion and integration are inadequate. To close this gap, it is essential to standardize data collection and reporting across both central and local government units. Additionally, greater investment is needed in technical capacity and research resources to ensure consistent implementation. Addressing these challenges requires focused efforts on improving system interoperability, promoting user engagement, and adapting digital tools to local adaptation, thereby maximizing their effectiveness in ASF prevention and control.

Underreporting and behavior-driven transmission risks: Although national policies emphasize compliance with biosecurity measures, diagnostic testing, and depopulation protocols, the non-reporting of ASF-suspect cases is an ongoing challenge. Expert estimates indicate underreporting rates of up to 80–90%, depending on farm type (19). This underreporting largely stems from the premature sale of pigs through middlemen, driven by a lack of economic incentives and the absence of compensation for affected farmers. Addressing these behavior-driven issues requires systematic and interdisciplinary research approaches, integrating economic modeling, social science, epidemiology, and policy analysis. Such efforts are essential to improve policy compliance at the community level, enhance local disease reporting incentives, and ultimately enable timely detection and effective outbreak response at different scales.

ASF vaccine procurement and rollout: Recent efforts by the BAI to procure and deploy ASF vaccines signal a promising shift in control strategy (22). However, success will depend on real-world field validation, clear guidelines for use across different types of farms and LGUs, and farmer confidence in both safety and effectiveness. Without regulatory clarity, premature rollout may undermine trust. Coordinated research and implementation frameworks are needed to ensure that vaccines enhance, not complicate, ASF control efforts.

Environmental consequences of ASF outbreaks: ASF’s impacts extend beyond animal health. The improper disposal of ASF-infected carcasses and waste, especially during the flooding season, can result in long-term contamination of water bodies, soil, and air, potentially contributing to further virus transmission to broader areas. Four Philippine endemic wild pig species (*Sus philippensis, S. cebifrons, S. oliveri, S. ahoenobarbus*), all listed as threatened on the IUCN Red List, may all be at risk of ASF infection (23, 24). While their precise role in ASF transmission remains unclear and differs from wild boar (*Sus scrofa*) in other countries, their conservation status highlights the need to integrate biodiversity considerations into ASF control strategies. These environmental and conservation impacts are likely underestimated in current assessments. Consequently, One Health-oriented research is urgently needed to evaluate these consequences and their broader implications for animal health, human well-being, and ecosystem integrity.

## Data Availability

The original contributions presented in the study are included in the article/Supplementary material, further inquiries can be directed to the corresponding author.
