# Supplementary material for: Perspective: challenges and research opportunities to enhance African Swine Fever control in the Philippines
Source: Front Vet Sci. 2025 Sep 30;12:1675095. doi: 10.3389/fvets.2025.1675095 (PMC12518057; doi:10.3389/fvets.2025.1675095)
Supplement: Supplementary file 1 [file Table_1.DOCX]

Challenges and research opportunities to enhance African Swine Fever control in the Philippines

Start of Block: MainBlock

Q1 Which Cohort

- Cohort 1 (1)
- Cohort 2 (2)
- Prefer not to say (3)

Q2 Rank the importance (9=Very Important; 1= Not important at all)

|  | 1 | 9 |
| --- | --- | --- |

| Difficult to get sample from backyard population () | 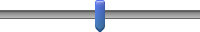 |
| --- | --- |
| Relevant local incentives to report the disease/ Underreporting / Policy Non-Compliance () | 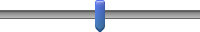 |
| Lack of timely information and data sharing between stakeholders. Digital Epidemiology Monitoring and the need to improve traceability. () | 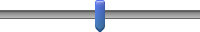 |
| Inconsistent enforcement of guidelines across LGUs () | 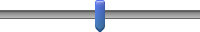 |
| Shifting from Epidemic to an Endemic Mindset () | 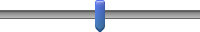 |
| Urban-Rural Inequality. Limited education and resources for some farmers. () | 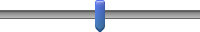 |
| Intermediaries (middleman) is an issue in swine supply chain () | 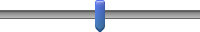 |
| Inappropriate Slaughtering Practices () | 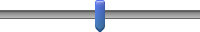 |
| Vaccine use (will it make ASF control easier or harder?) () | 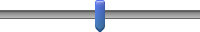 |
| others () | 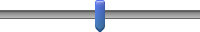 |

Q2 What do you mean by "others"? (you can provide new issues or N/A)

________________________________________________________________

End of Block: MainBlock
